# Factors affecting treatment-seeking for febrile illness in a malaria endemic block in Boudh district, Orissa, India: policy implications for malaria control

**DOI:** 10.1186/1475-2875-9-377

**Published:** 2010-12-30

**Authors:** Ashis Das, TK Sundari Ravindran

**Affiliations:** 1 Independent Public Health Researcher, Bhubaneswar, 751012, India; 2 Achutha Menon Center for Health Science Studies, Sree Chitra Tirunal Institute for Medical Sciences and Technology, Trivandrum, 695011, India

## Abstract

**Background:**

Orissa state in eastern India accounts for the highest malaria burden to the nation. However, evidences are limited on its treatment-seeking behaviour in the state. We assessed the treatment-seeking behaviour towards febrile illness in a malaria endemic district in Orissa.

**Methods:**

A cross-sectional community-based survey was carried out during the high malaria transmission season of 2006 in Boudh district. Respondents (n = 300) who had fever with chills within two weeks prior to the day of data collection were selected through a multi-stage sampling and interviewed with a pre-tested and structured interview schedule. Malaria treatment providers (n = 23) were interviewed in the district to gather their insights on factors associated with prompt and effective treatment through a semi-structured and open-ended interview guideline.

**Results:**

Majority of respondents (n = 281) sought some sort of treatment e.g. government health facility (35.7%), less qualified providers (31.3%), and community level health workers and volunteers (24.3%). The single most common reason (66.9%) for choosing a provider was proximity. Over a half (55.7%) sought treatment from appropriate providers within 48 hours of onset of symptoms. Respondents under five years (OR 2.00, 95% CI 0.84-4.80, *P *= 0.012), belonging to scheduled tribe community (OR 2.13, 95% CI 1.11-4.07, *P *= 0.022) and visiting a provider more than five kilometers (OR 2.04, 95% CI 1.09-3.83, *P *= 0.026) were more likely to have delayed or inappropriate treatment. Interviews with the providers indicated that patients' lack of trust in community volunteers providing treatment led to inappropriate treatment-seeking from the less qualified providers. The reasons for the lack of trust included drug side effects, suspicions about drug quality, stock-outs of drugs and inappropriate attitude of the provider.

**Conclusion:**

Large-scale involvement of less qualified providers is suggested in the malaria control programme as volunteers after appropriate capacity development since the community has more trust in them. This should be supported by uninterrupted supply of drugs to the community volunteers, and involvement of the community-based organizations and volunteers in the planning, implementation, and monitoring of malaria control services. There is also a need for continuous and rigorous impact evaluations of the program to make necessary modifications, scale up and to prevent drug resistance.

## Background

Malaria is one of the world's major public health concerns, contributing to 243 million clinical cases and under a million deaths each year. For centuries, malaria has impaired productivity, economic growth, child development and learning, and health status on a large scale. The disease also takes a high toll on households and health care systems. As per the estimates of the World Health Organization, malaria reduces GDP growth by approximately one percentage point per year [1].

India leads the South East Asia region in terms of malaria cases and ranks second only to Myanmar in the number of officially reported deaths attributed to malaria [2]. Almost 95% of the country's population lives in malaria-risk areas and 80% of the cases are reported from 20% of the population in the country. Five states account for 60% of malaria cases: Orissa, Chhattisgarh, Madhya Pradesh, Jharkhand and West Bengal [3]. Orissa, a state on the eastern coast contributes to the maximum number of malaria cases and deaths in the country. With 3.8% of India's population, Orissa accounted for one-fourths of total malaria cases, one-fifths of malaria deaths and 42% of *Plasmodium falciparum *infection of the national malaria burden in 2008 [4].

Roll Back Malaria (RBM) Partnership, an initiative jointly launched by WHO, UNICEF, UNDP and the World Bank, has pledged to reduce the numbers of malaria cases and deaths recorded in 2000 by 50% or more by the end of 2010 and by 75% or more by 2015 through the Global Malaria Action Plan (GMAP) [5]. Early diagnosis and effective treatment are among the principal strategies for control of malaria. Prompt and appropriate treatment is essential to reduce the severity and complexity of the disease, and minimize economic burden and long-term adverse effects on the health system [6]. Orissa state accounts for a substantial malaria burden to India. It has a largely difficult terrain, remote and under served areas, along with 40% of population below the poverty line and 22% scheduled tribe populations, calling for special attention on early treatment. Prompt treatment depends on early recognition of the disease symptoms followed by timely care-seeking from appropriate health care providers. However, evidence on treatment-seeking behaviour in the state is scanty.

Treatment-seeking behaviour was assessed among persons affected by febrile illness in a malaria endemic district in Orissa during the high malaria transmission season.

## Methods

### Study setting

**Figure 1:**
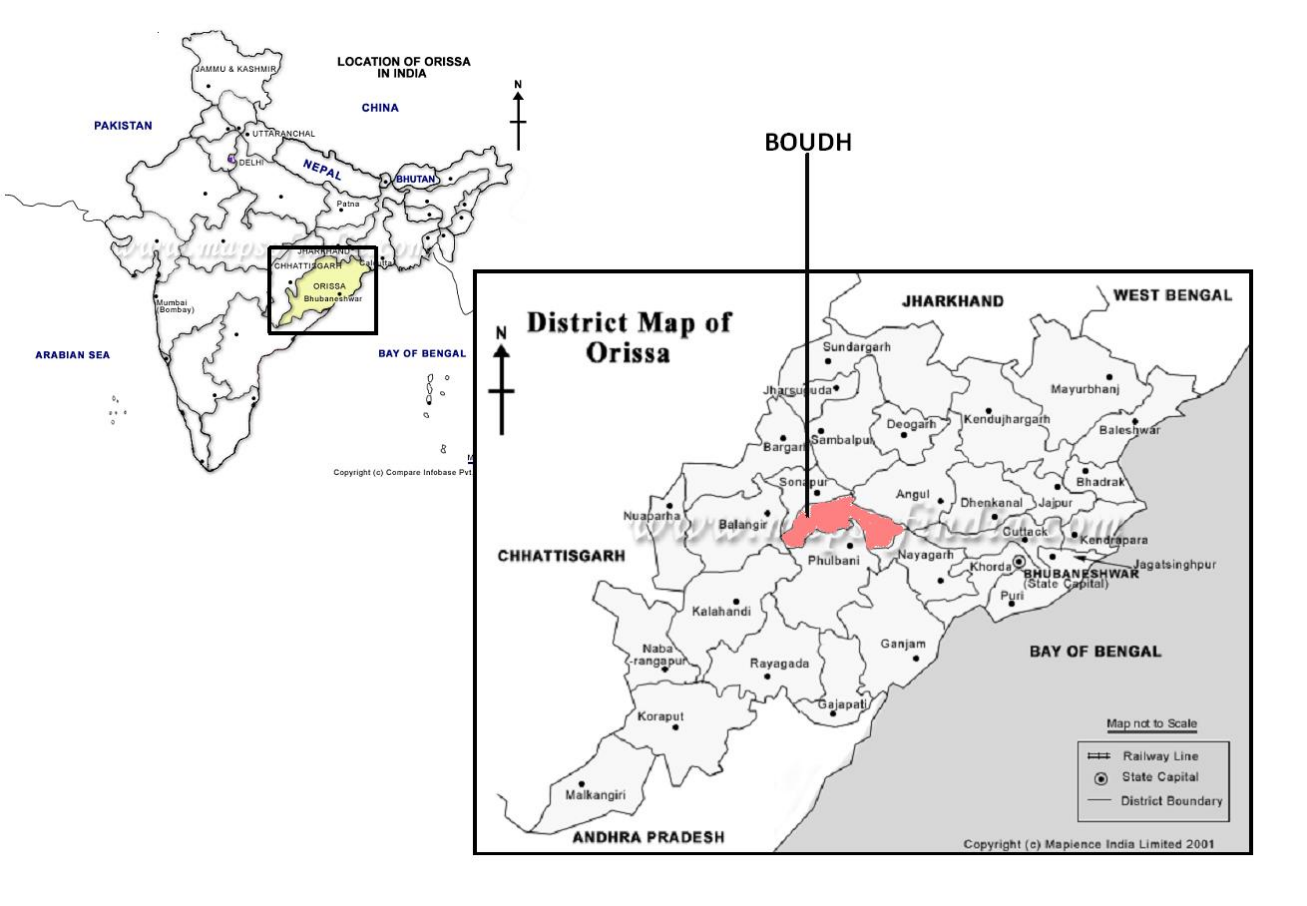
**Location of Boudh district, Orissa state, India**. A map showing the location of the study district Boudh in Orissa state of India.

The study was conducted in Boudh, a central district in Orissa bound by the rivers Mahanadi and Tel (Figure 1). It extends over an area of 3444.70 sq. km. with forests covering 32% of the total area. The district has 373,372 inhabitants, constituting 1.02% of the state's total population. The urban inhabitants are only 5% of the population, whereas indigenous population consists of 13% in the district. Agriculture stands as the major economic activity in the district. The adult literacy rate is 58.43% [7]. The next administrative unit below a district is known as a community development block or in short "block". The district has three administrative blocks with each block having a block primary health center (BPHC). Each BPHC caters to a population of 80,000 to 120,000. It provides curative and preventive services at the block level. The BPHC is managed by specialists (medicine, paediatrics, surgery, obstetrics and gynecology) doctors and paramedical staff, who provide inpatient and outpatient services. Each BPHC's catchment area is further sub-divided into three sectors, each having one sector primary health center. The sector PHC is run by a general physician and two to three paramedical staff. The basic unit of health service provision at the community level is called a sub health center. A sub health center is managed by two (one male and one female) health workers and it caters to 3,000 to 5,000 population.

As per the data from the State Malaria Office, Boudh reported 40,084 fever cases, 3,572 malaria cases and 26 deaths due to malaria in 2005. Annual parasite incidence (malaria incidence as confirmed by microscopy per 1,000 population) was 9 and falciparum malaria constituted 60% of all malaria cases. Boudh ranked 15^th ^in the state among all the 30 districts in terms of malaria incidence in that year (Personal communication: State Malaria Office, Orissa). The district shares similar features with most of the malaria endemic districts, e.g. high incidence of malaria, forested and hilly areas, and presence of tribal population.

### Study design and sampling

A cross-sectional community-based survey was carried out during the high malaria transmission season (June - August) of 2006. The respondents were selected through a multi-stage sampling. In each stage, the sampling units were selected based on the criteria of maximum morbidity and mortality due to malaria in the previous year. One of the block primary health centers (BPHC) was selected out of three in the first stage; three sector primary health centers (PHC) under this BPHC in the second stage; one health sub-center within each PHC in the third stage and considered each health sub-center area as a cluster. In the fourth stage, three to five villages were selected within each cluster at random and line-listed the households to identify cases with fever and chills within the previous two weeks of data collection. We selected equal number of subjects from each of these three clusters.

Only those persons who reported (or for whom caretakers reported) fever with chills within two weeks prior to the day of data collection were interviewed. The inclusion of chills along with fever was based on the assumption that since chills is a common symptom of malaria, taking both the symptoms would narrow down the case definition and might exclude some of the false positives in the sample as compared to taking fever cases alone. Persons who were sick during the data collection were excluded for ethical reasons. Infants and pregnant women were also excluded assuming that care-seeking for these groups would be different compared to others in the community. With the assumptions of prevalence of fever 10% (based on the available literature in similar settings), precision 5%, and 95% CI, STATCALC module of Epi Info software estimated the required sample size to be 138, which was doubled and rounded off it to 300 to minimize the design effect of a multistage sampling [8,9]. Equal numbers of subjects (100 each) were interviewed in each of the three clusters.

Malaria treatment providers (n = 23) were purposively selected based on their knowledge and role in malaria control, provision of services in the study area, and caseload. They were district malaria officer (n = 1), PHC Medical Officers (n = 3), malaria laboratory technician (n = 1), male health workers (n = 3), female health workers (n = 3), drug distribution center (DDC) holders (n = 6), and less qualified informal service providers (n = 6).

### Data collection and analysis

Primary information was collected from the respondents with the help of a pre-tested, structured and closed-ended interview schedule, translated in Oriya. The respondents were asked about their health care-seeking behaviour with respect to the recent episode of the disease and the actions they took for their recent episode of fever, pathways of treatment seeking, reasons for such actions, distance and mode of transport to the provider. Reasons for not going to a provider appointed by the government health system were explored. In-depth interviews with the providers were carried out using a semi-structured and open-ended interview guideline in a tape recorder. They were asked their opinions about the factors affecting treatment-seeking for febrile illness in the local population. Each interview lasted from 20 to 45 minutes.

The information from questionnaire survey was entered in Microsoft Excel and later analysed with SPSS version 14. Univariate, bivariate and multivariate analyses were undertaken and Chi-square 'p' value of < 0.05 was considered statistically significant. The recorded interviews were transcribed verbatim on the same day of interview. The transcripts were then coded and the codes were grouped into themes. The themes were analyzed manually to observe for any particular trend.

Malaria treatment providers in the district were interviewed to gather their insights on factors associated with prompt and effective treatment. The interviews were carried out using a semi-structured and open-ended interview guideline.

### Ethical considerations

Respondents were informed of the objectives of the study and the use of information they would be giving and collected their signature or thumb impression on the informed consent form. The data were coded and the identities of the respondents were kept completely confidential. The Institutional Ethics Committee of Sree Chitra Tirunal Institute for Medical Sciences and Technology, Trivandrum, Kerala gave the ethical clearance for the study (approval number IEC/120/May 22, 2006).

### Definitions

Promptness and effectiveness of treatment were assessed. Promptness meant a treatment, which was availed within 48 hours of developing symptoms. This benchmark was based on a similar study carried out in the South Asian context by Reilly *et al *[10]. Effective treatment was defined as management of cases with anti-malarial drugs as per the national malaria control programme protocol and administered by a provider who is designated by the programme. During the study, as per the guidelines of the National Vector Borne Diseases Control Programme (NVBDCP) in India, radical treatment for malaria was available at the peripheral health facilities like primary health centers. Health sub centers and village level drug distribution centers (DDC) managed by volunteers selected by the programme were providing presumptive treatment. Presumptive treatment consisted of four tablets of chloroquine, and radical treatment consisted of the full course of ten chloroquine tablets. Thus, a person availing treatment from an NVBDCP designated provider within 48 hours of developing the symptoms was classified as receiving "prompt and effective treatment". Any deviation from this was classified as "delayed and ineffective treatment", i.e. treatment availed beyond 48 hours of developing the symptoms and/or treatment by any other service provider other than those designated by the NVBDCP.

For this study, less qualified providers (LQP) were defined as informal healthcare providers, who prescribe allopathic medicines for different diseases. These LQPs had never undertaken any course or training, which would legally enable them to prescribe allopathic medicines for malaria or any other disease. They had also not been trained on malaria treatment.

## Results

### Sample characteristics

As shown in table 1, 300 respondents were interviewed out of which 180 (60%) were men and 120 (40%) were women. The mean age for the sample was 25.7 years. Majority of the respondents were above 14 years and scheduled caste was the dominant caste group in the sample. More than half of the respondents had at least one year of schooling, with a mean of 2.59 years. About double men (72.8%) than women (35.8%) had at least one year of schooling. The single largest occupation group was of students followed by farmers and daily-wage laborers. The standard of living index was calculated by using the standard of living (SLI) matrix of National Family Health Survey 2 with slight modification for the rural population [11]. The distribution showed that about half of the study population had low standard of living.

**Table 1:** Sample characteristics

Variable	Men (%) (n = 180)	Women (%) (n = 120)	Total (%) (n = 300)
**Age**			
< 5 years	28 (15.6)	15 (12.5)	43 (14.3)
5-14 years	51 (28.3)	30 (25)	81 (27)
> 14 years	101 (56.1)	75 (62.5)	176 (58.7)
Mean (SD)	24.98 (20.44)	26.68 (19.03)	25.66 (19.87)
**Caste**			
Scheduled Caste ^a^	74 (41.1)	55 (45.8)	129 (43)
Scheduled Tribe ^b^	30 (16.7)	26 (21.7)	56 (18.7)
Others	76 (42.2)	39 (32.5)	115 (38.3)
**Education (Years of schooling)**			
Non-literate (0)	49 (27.2)	77 (64.2)	126 (42)
Primary school (1-5)	88 (48.9)	33 (27.5)	121 (40.3)
High school and above (>6)	43 (23.9)	10 (8.3)	53 (17.7)
Mean (SD)	3.36 (3.03)	1.44 (2.40)	2.59 (2.95)
**Occupation**			
Farmer	44 (24.4)	18 (15.0)	62 (20.7)
Trader	3 (1.7)	0 (0)	3 (1)
Daily-wage laborer	38 (21.1)	22 (18.3)	60 (20)
Homemaker	0 (0)	33 (27.5)	33 (11.3)
Student	55 (30.6)	24 (20)	79 (26.3)
Not working	40 (22.2)	23 (19.2)	63 (20.7)
**Standard of Living Index**			
Lower (0-14)	89 (49.4)	68 (56.7)	157 (52.3)
Middle (15-24)	85 (47.2)	50 (41.7)	135 (45)
Higher (25-67)	6 (3.3)	2 (1.7)	8 (2.7)

a - socio-economically marginalized community, given special focus and privileges by the Government of India

b - socio-economically marginalized tribal population, given special focus and privileges by the Government of India

### Treatment-seeking behaviour

The vast majority (n = 281) sought treatment. Nine respondents (six women and three men) did not take any action, and 10 (six women and four men) adopted self-medication. A little over a third (37%) went to a government health center for treatment, and about a fourth (24.3%) sought treatment from a community health volunteer. However, a third of those with fever and chills (32.3%) went to a less qualified provider (LQP) for treatment despite the presence of community health volunteers (DDC) appointed by the NVBDCP (table 2).

**Table 2:** Treatment-seeking behaviour for the last episode of febrile illness

First actions	Men (%)	Women (%)	Total (%)
Government health center	63(35)	44(36.7)	107(35.7)
Less qualified provider	69(38.3)	28(23.3)	97(32.3)
Community level volunteer (DDC)	39(21.7)	34(28.3)	73(24.3)
Self-medication	4(2.2)	6(5)	10(3.3)
Homeopath	2(1.1)	1(0.8)	3(1)
Traditional healer	0(0)	1(0.8)	1(0.3)
Did not take action	3(1.7)	6(5)	9(3)
Total	180	120	300

Reasons for choice of provider*	**Men (%)**	**Women (%)**	**Total (%)**

Proximity	122(70.5)	66(61.1)	188(66.9)
Low cost of care	24(13.9)	17(15.7)	41(14.6)
Faith on provider	17(9.8)	13(12)	30(10.7)
Provider attitude and availability	8(4.6)	10 (9.1)	18 (6.4)
Payment in credit	2(1.2)	2(1.9)	4(1.4)
Total	173	108	281

Distance*	**Men (%)**	**Women (%)**	**Total (%)**

≤ 5 KMs	151 (87.3)	82(75.9)	233 (82.9)
> 5 KMs	22 (12.7)	26 (24.1)	48 (17.1)
Total	173	108	281
Mean (SD)	3.45 (5.98)	5 (7.99)	4.05 (6.86)

Transport*	**Men (%)**	**Women (%)**	**Total (%)**

Walking	76(43.9)	57(52.8)	133 (47.3)
Bicycle	85(49.1)	37(34.3)	122(43.4)
Public transport	10(5.8)	12(11.1)	22(7.8)
Others (bullock cart, motorbike, rented vehicle)	2(1.2)	2(1.9)	4(1.4)
Total	173	108	281

* We have excluded respondents with self-treatment or no treatment at all (n = 19).

Two-thirds stated proximity as the reason for choosing the provider for their last episode of febrile illness (fever and chills), whereas others preferred their provider because of the low cost, confidence, availability and attitude of the provider (table 2).

The mean distance from the first health care provider was 4.05 kilometers, with women (5 KM) travelling farther than men (3.45KM). More women than men visited the district headquarters hospital situated at a distance of more than 50 kilometers as they had severe illness, which resulted in more mean distance for women. Forty seven percent of respondents walked to their providers, whereas others took bicycle, public transport, and other means of transport (table 2).

### Determinants of prompt and effective treatment

More than half (55.7%) of respondents had availed prompt and effective treatment, i.e. availing treatment within 48 hours of onset of symptoms from a provider who has been designated by the NVBDCP to provide malaria treatment (table 3). From the multiple logistic regression analysis, age, caste and distance from the provider were found to be independently associated with prompt and effective treatment (table 4). For example, children under five years of age had the least prompt and least effective treatment among all age groups. The highest proportion of those receiving treatment belonged to scheduled caste, with the least from the scheduled tribes group. A higher proportion of those with schooling had received treatment as compared to those who had never been to school. More respondents who availed care from a provider less than 5 KM received prompt and effective treatment. Respondents under five years (OR 2.00, 95% CI 0.84-4.80, *P *= 0.012), belonging to scheduled tribe community (OR 2.13, 95% CI 1.11-4.07, *P *= 0.022) and visiting a provider more than five kilometers (OR 2.04, 95% CI 1.09-3.83, *P *= 0.026) were two times more likely to have delayed and/or ineffective treatment than their counterparts. There was no association between gender and appropriate treatment-seeking behaviour.

**Table 3:** Association of prompt and effective treatment with predictor variables

Variables	Prompt and effective treatment n (%)	Delayed and ineffective treatment n (%)	Total n	P
**Sex**				
Men	97 (53.9)	83 (46.1)	180	0.261
Women	70 (58.3)	50 (41.7)	120	
**Age group**				
< 5 years	19 (44.2)	24 (55.8)	43	** 0.020**
≥ 5 years	148 (57.6)	109 (42.4)	257	
**Caste**				
Scheduled Tribe	24 (42.9)	32 (57.1)	56	** 0.038**
Scheduled Caste	81 (62.8)	48 (37.2)	129	
Others	62 (53.9)	53 (46.1)	115	
				
**Education**				
No schooling	60 (47.6)	66 (52.4)	126	**0.017**
Any schooling	107 (61.5)	67 (38.5)	174	
**Standard of living index**				
Lower	87(55.4)	70(44.6)	157	0.925
Middle	75(55.6)	60(44.4)	135	
Higher	5(62.5)	3(37.5)	8	
Distance travelled for care^*^				
≤ 5 KMs	143 (61.4)	90 (38.6)	233	** 0.024**
> 5 KMs	21 (43.8)	27 (56.3)	48	

* We have excluded respondents with self-treatment or no treatment at all (n = 19).

**Table 4:** Predictors of prompt and effective treatment

Predictor variable	Odds ratio	95% CI	P
Age group (years)			
< 5§	1.00	__	__
≥ 5	2.00	0.84-4.80	0.012
Caste			
ST§	1.00	__	__
SC	2.13	1.11-4.07	0.022
Others	1.61	0.94-2.76	0.081
Education			
No schooling§	1.00	__	__
Some schooling	1.27	0.72-2.25	0.402
Distance (kilometers)			
≤ 5§	1	__	__
> 5	2.04	1.09-3.83	0.026

§ Reference group

### Factors affecting prompt and effective treatment: providers' perspectives

#### Negligence

In providers' opinion negligence by those developing symptoms stood out to be the single most common (n = 12) factor underlying delayed and/or ineffective treatment. Although community drug distribution centers (DDC) existed in almost all localities to provide free presumptive treatment of malaria with four tablets of chloroquine, those with symptoms did not take medicine at the appropriate time. They would carry on with their daily routine and delay treatment till they were unable to function any longer. In the case of children, since older children took care of most of the younger children, their inability to recognize the malaria symptoms led to delayed care for most of the children. Women were also regular victims of inappropriate care as they tended to neglect their health due to household work and sometimes, because there was no one to escort them to the health center.

*"First day they wait for the symptoms to subside attributing these to common cold, then they don't get time from their daily routine to go to the health care provider, gradually 4 or 5 days would pass. Most common victims of negligence are ladies which they attribute to their household chores, sometimes they would not have anybody to accompany to the PHC." *[PHC medical officer]

#### Noncompliance

The providers (n = 10) said most people did not take the drugs as prescribed. Though four cloroquine tablets were provided as one time dose (for presumptive treatment), patients would take one or two at a time till they got symptomatic relief and preserved the remaining tablets for future use. Though the tablets gave temporary relief for a few days, the fever often came back. Since most of the people are poor, they cannot stay back at home when sick and lose their daily wages.

*"People don't take the complete dose since symptoms relieve after taking one or two tablets."*[Female Health Worker]

According to some providers (n = 7), noncompliance comes out of previous experiences of side effects of the prescribed drugs. They complain of dizziness, vomiting, loss of appetite and for children bitter taste (chloroquine) and some are unhappy over too many tablets. For an adult the usual dosage (radical treatment) is ten tablets of chloroquine and six tablets of primaquine, which has to be taken over a period of three days. Sometimes, they do not have anything to eat as they remain at home due to sickness. However, anti-malarial drugs, especially chloroquine gives acute side effects if taken in empty stomach.

#### Availing treatment from less qualified providers

If there is no cure after presumptive treatment, they go to less qualified providers who apply various measures like injecting analgesics, antibiotics, even sedatives. These providers have some advantages such as their availability where there is no doctor and flexibility for the people to pay the fees in installments or in kind. At times, people consume DDC supplied chloroquine while availing treatment from the less qualified providers simultaneously. Though they get cured due to chloroquine, they attribute their cure to the less qualified providers' treatment.

*"They (less qualified providers) do not provide proper treatment at all. They administer whatever they feel appropriate. For two persons presenting with the same symptoms they would prescribe different medicines." *[Male health worker].

While patients fail to get cured due to non-compliance or incomplete treatment, they attribute it to the lack of efficacy of drugs provided by the government programmes. The less qualified practitioners reinforce their lack of faith in government drugs through misinformation.

*"Most of them don't take the medicine as prescribed. They discontinue from the regimen as symptoms are relieved. If they get fever again within a short span of time, they attribute it to the ineffectiveness of the medicines we supply." *[Drug distribution center]

*"The free drugs supplied by the government are not able to cure people. How could you expect the free drugs to match the efficacy of the drugs I sell in my medicine store?" *[Less qualified provider]

Previous experiences of ineffective treatment from the DDCs, drug side effects, and pressure to get back to work early drive patients to seek quick relief with a single shot of medicine or injections. Taking advantage of the prevailing perception, less qualified providers indulge in irrational and inappropriate treatment by enticing the patients with quick relief through expensive antibiotic injections.

*"About 60% people demand injection due to bitter taste (children), dizziness, reeling of head (adults) associated with chloroquine. They say that these tablets will not cure me. Please give me a 'suja' (injection)." *[PHC medical officer]

*"People want quick relief. They do not want to take medicines for days. Even if this treatment is expensive, they go for it. In this way they perceive injection to be most appropriate." *[Female health worker]

*"People insist on Injection- as tablet causes nausea, loss of appetite, vomiting, head reeling." *[Less qualified provider]

#### Drug distribution center

As per the norm, there is one DDC per 1,000 population. If villages are small, one DDC is shared among two or three villages. In this case, patients in those villages where the volunteer does not stay face difficulties in accessing timely care during odd hours. At times, people of the other villages do not know about the facilities available at DDC. Moreover, there have been instances of repeated drug stock-outs compelling the patients to seek care from the informal sector.

*"At places we have one DDC per three to five small tribal hamlets which would be placed 5 to10 kilometers apart. If somebody develops fever at night, it becomes virtually impossible to go to the DDC among dense forest to get medicine." *[District Malaria Officer]

*"Our village is far off from the primary health center. Whenever we have stock-outs, we wait for the male health worker to replenish the stocks. Sometimes it takes days to get the medicines and if any fever patient comes to us during that time, we send them back." *[Drug distribution center]

## Discussion

Early recognition of malaria symptoms like fever and subsequent treatment with an effective anti-malarial is essential to reduce morbidity, mortality and complications due to malaria. Better understanding of community's treatment-seeking behaviour is crucial for the disease control programs to plan and implement, and utilize available resources efficiently. The present study in Boudh district in eastern India documents that children less than five years, indigenous communities and communities residing far from the health providers and facilities are at a higher risk of not availing timely treatment for febrile illness. Only about 60% of the respondents received timely and appropriate treatment. Communities have more trust on less qualified providers than village level drug distribution centers managed by volunteers.

Delay in receiving treatment for malaria can be fatal. A study in West Bengal has shown a delay of more than 48 hours from the onset of treatment to be a determinant of fatality [12]. There are a few studies in Orissa setting where delay in onset of treatment and presence of complications on admission were found to be associated with mortality [13,14]. The duration of care-seeking for the majority of the subjects was much quicker than that of others in similar Indian settings [15,16]. Nevertheless, this falls short of the recommended timeline by the Roll Back Malaria [5]. Most of the providers commented that negligence by the patients was the primary reason for delay. However, most of the residents in the study area were dependent on daily earnings for their subsistence e.g. daily wage laborers or small-scale farmers. There is a possibility of ignoring the symptoms until the person is seriously ill, if his/her family depends on the daily earnings.

More symptomatic subjects had accessed prompt and effective treatment compared to studies conducted in similar settings except one conducted in urban Gujarat [16]. Though the study was conducted in a rural area with a fifth of the population from the indigenous tribal community, availing services from traditional healers was very low compared to similar studies [17] However, care-seeking from LQPs was higher than other Indian settings [15,16,18,19].

Distance from the first health care provider came out to be significant in the study through multivariate analysis after adjustment with age of the respondents and caste. Distance to the health care provider has already been substantiated by many studies and this study adds to earlier literature [20-23]. However, in this case it was not the actual distance to the nearest appropriate provider or facility that led to delay in treatment-seeking. Rather it was the perception of not acknowledging the nearest provider (DDC managed by a community volunteer) as a suitable provider due to various reasons. This lack of community trust on the DDC has been shown by another study conducted in similar settings [24]. One of the reasons came out from the exploratory qualitative study was the perception of relative ineffectiveness of the drugs distributed free of cost by the DDC than the drugs available in the market. In addition, there were occasional stock-outs of drugs, which would have compelled the patients to seek care from elsewhere, mostly the informal service providers. Repeated stock-outs of drugs from a DDC in a village might lead to regular treatment-seeking from LQPs. On the other hand, there are multiplicities of factors playing role for the acceptability of the less qualified providers. They not only stay nearby, they also provide treatment whenever there is a need and sometimes the clients have the option to pay later. This calls for large-scale inclusion of less qualified informal service providers into formal health system after adequate capacity development and further supervision and monitoring.

The study was carried out in an era when the National Vector Borne Diseases Control Programme recommended chloroquine as the first-line drug for treatment of malaria. Community level health workers and volunteers managing drug distribution centers (DDC) were providing presumptive treatment at the village level. Now, artemisinin-based combination therapy (ACT) has been introduced for falciparum malaria in areas of chloroquine resistance, whereas chloroquine continues to be the drug of choice for vivax malaria and in places where the disease is still sensitive to it. However, despite the change in regimen, the anti-malarials are being provided at the community level by a cadre of community health volunteers known as ASHA (Accredited Social Health Activist). Community level management of fever by ASHA is being rolled out in all the high burden districts in India [25]. The study findings are relevant in the context of implementing ACT, as the community may continue to discard the remaining tablets after receiving symptomatic relief and non-adherence to the regimen persists. Chloroquine resistance in many places has been attributed to incomplete dosage [26]. Emergence of resistance to ACT in a similar fashion will be a serious public health challenge in a resource poor health system setting like Orissa. Similarly, the supply of drugs to the ASHA has to be regular to prevent any stock-outs and subsequent community's loss of faith in her, which might lead to care-seeking from less qualified providers.

Malaria contributes to about 5% of deaths in children in South Asia, but is a serious contributor to morbidity and chronic anaemia [27]. A study in Bangladesh has shown higher rates of malaria prevalence in children, whereas another in the neighbouring state West Bengal showed highest case fatality rate among children under five [12,28]. Children under five years are found to be more vulnerable to malaria. Most malaria deaths among young children occur within 2-3 days after onset of symptoms [29]. However, in this study less than half of children under five had sought treatment within 48 hours of onset of symptoms. Some of the providers opined the delay in access to appropriate treatment is due to their parents' negligence. The reasons for negligence could be an interplay of ignorance, lack of economic access, or providers' attitude. Access to treatment and treatment-seeking behaviour of malaria for children under five is a less studied domain in South Asia and it calls for generating more evidences in this domain.

The tribal community was found to be more vulnerable for appropriate and timely treatment for fever. Inappropriate care-seeking by the tribals might be due to their low level of awareness about symptoms and seriousness of malaria, and availability of services by the public health system. Majority of India's indigenous population lives in areas with high malaria endemicity. Even though the tribal communities are only 8% of the total population, they contribute 30% of total malaria cases, 60% of total *Plasmodium falciparum *cases and 50% of malaria deaths [30]. More than 60% of tribal population of Orissa lives in high-risk areas for malaria [31]. The areas inhabited by the tribal communities are forested, hilly terrain, hard-to-reach, and penetration of the public health system is poor. They are diverse culturally and linguistically. That is why most of the conventionally designed public health campaigns and services fail to alter their behaviour. This calls for change in the mode of delivery of health services and campaigns by using more culturally appropriate media, local dialects, and involving change agents from their own communities.

Similarly, community ownership of public health programmes has remained very low in this region. Taking inspiration from successful examples of community based malaria control programs across the world, arguments are made for implementing such programmes in places similar to the study area where people can manage and monitor by taking ownership of the programme [32-34]. With the introduction of village health and sanitation committees (VHSC) by the National Rural Health Mission (NRHM) in India, there are many avenues to involve the community in the decision making, planning, implementation and monitoring of public health programs including control of vector borne diseases [35]. Involving the VHSC will also provide an opportunity to integrate malaria control within the frameworks of primary health care and make it sustainable since the committees receive annually an untied fund for their activities. ASHA is an integral part of the VHSC. Adequate capacity development of VHSC will give a scope for monitoring ASHA's performance at the local level. There have also been successful examples of community groups led by women involved in primary health care. Women can play a more active role in the health programs and welfare of their communities if they are handed over the responsibility to transmit knowledge about all aspects of malaria and raise awareness about the facilities available in their community for appropriate treatment-seeking. In this context, it is argued to involve various self-help groups (SHGs) in the community [36].

The findings in this study are based on self-reported symptoms. There might be issues of recall and reporting bias. However, attempt was made to minimize such biases by translation and back-translation and piloting of the schedule before actual administration to make it more context specific, and reducing the recall period to two weeks. Nevertheless, in real life scenario, it is the patient's or the caregiver's perception of illness which initiates treatment-seeking, rather than a clinical or laboratory diagnosis. Secondly, at the time of study, the national malaria control guidelines recommended all cases of fever in endemic areas to be tested for malaria and treated accordingly. The selection of cases from high endemic clusters at each stratum would have introduced selection bias. However, in a block with a homogenous population distribution and health infrastructure, the health-seeking behaviour is not expected to vary much.

## Conclusion

The treatment-seeking behaviour was studied among persons affected by febrile illness in a malaria endemic district in Orissa during the high malaria transmission season. The indigenous populations and children under five years are less likely to receive timely and appropriate treatment for fever. The community has more faith on the less qualified providers despite their irrational treatment and less trust on the community volunteers even though they provide drugs as per the national guidelines. The following suggestions are recommended; 1) mainstreaming of less qualified providers in the malaria control programme, 2) uninterrupted supply of drugs and supplies to the community volunteers, 3) involvement of the community-based organizations and volunteers in the planning, implementation, and monitoring of malaria control services. There is a need of continuous and rigorous impact evaluations of the programme to make necessary modifications, scale up and prevention of drug resistance.

## Competing interests

The authors declare that they have no competing interests.

## Authors' contributions

Both the authors conceived the study, participated in its design and drafted the manuscript. All authors read and approved the final manuscript.
